# Beyond protection: the growing role of instrumented mouthguards in sports

**DOI:** 10.1038/s41415-026-9547-4

**Published:** 2026-02-27

**Authors:** John Patrick Haughey, Gregory Tierney, Garreth Farrell, Ian Needleman

**Affiliations:** 168647973607245053001 Eastman Dental Institute, Department of CPD, University College London, London, United Kingdom; 762271836157107016211 School of Sport, Ulster University, Belfast, United Kingdom; 706715944742886566999 Leinster Rugby, Dublin, Ireland; 098629276772306839797 Eastman Dental Institute, Periodontology, University College London, London, United Kingdom

## Abstract

Instrumented mouthguards (iMGs) are being used in sport to help with sport-related concussion management, performance optimisation and injury prevention. iMGs represent a significant advancement at the intersection of sports dentistry, biomechanics, and wearable technology. Evolving from traditional protective mouthguards, iMGs integrate sensors that can measure head acceleration events, providing real-time biomechanical data during training and competition. These devices have gained substantial attention due to their potential to support concussion protocols, monitor athlete performance, and aid in injury prevention. World Rugby's 2024 mandate requiring iMG use in elite-level competition marks a pivotal shift in sports safety standards, setting a precedent for broader adoption. iMGs offer several benefits, including accurate impact measurement, data-driven injury risk assessment, and support for return-to-play decisions. Despite their advantages, challenges such as cost, data interpretation, data accuracy, technology malfunction and wearer comfort remain. Design improvements, such as the miniaturisation and strategic placement of components, have addressed many early limitations. As sensor technology continues to evolve, iMGs are expected to integrate additional features and become more widely accessible, potentially extending their use to youth and amateur sports. This review explores the development, functionality, applications, and future potential of iMGs, highlighting their growing role in enhancing player safety and performance across high-impact sports.

## Key points

- Instrumented mouthguards (iMGs) integrate sensors that can measure head acceleration events, providing real-time biomechanical data during training and competition.
- The challenges involved with the management of concussion has led to the recent growth in iMG use.
- In 2024, World Rugby mandated instrumented iMG use in elite-level competition.

## Introduction

Mouthguards are widely used in sports to protect the teeth and mouth during training and competition. Recent developments have led to the integration of technology within the mouthguard, creating what are known as instrumented mouthguards (iMGs). These enhanced devices go beyond basic protection, offering real-time data collection and analysis. The development of iMGs is an interdisciplinary advancement, combining engineering, biomechanics, and sports dentistry.

The role of iMGs in assisting with sport-related concussion (SRC) management has caused a growing interest and rapid development in this technology in recent years. World Rugby's decision to make iMG wear mandatory for elite players during competition from 2024 has created a lot of attention.

This review explores how iMGs have evolved, the technology behind them, their benefits, current applications, challenges and limitations, and their future possibilities.

## The evolution of instrumented mouthguards

Mouthguards were first used in boxing in the early 20^th^ century to protect against orofacial injuries. By the 1950s, their use expanded to other contact sports, such as football, hockey, and basketball. Mouthguards were primarily designed to minimise physical injuries like tooth fractures, cuts, and jaw injuries. This protective role of mouthguards in preventing dental injuries is well-documented, with studies showing a significant reduction in dental trauma in athletes who wear them.^1^

Protective mouthguards are prominently made from ethylene-vinyl acetate (EVA),^2^ with a custom-fitted multi-layer laminated pressure thermoformed mouthguard being the recommended standard.^3^ Recent digital dentistry developments have seen a growing trend in 3D-printed protective mouthguards.^4^

The concept of using a mouthguard to house technology has led to the introduction of iMGs. Technology has been integrated into oral appliances for a range of measurable outcomes. These include the assessment of glucose in saliva, assessing saliva pH and biomarkers, monitoring respiratory rate, monitoring wellness, sleep electrooculography monitoring, detection of dehydration and physiological stress, teeth clenching and measuring sports head acceleration events (HAEs).^5^^,^^6^^,^^7^^,^^8^^,^^9^^,^^10^^,^^11^^,^^12^^,^^13^

The importance of understanding concussion and the challenges involved with the management of concussion has led to the recent growth in iMG use. The number and magnitude of HAEs can be assessed *in vivo* using inertial sensors to characterise the exposure in various sports and to help understand their potential relationship to concussion. Measuring devices include instrumented helmets, headbands, skin patches, mouthguards and earplugs.^14^

The development of the maxillary iMG for sports-related concussion management has seen their recent widespread use in elite-level contact sports. These devices combine the physical protective functions of traditional mouthguards with sensors that capture biomechanical data such as impact forces, accelerations, and rotational forces during athletic performance.

The maxillary mouthguard has been identified as the best position to use the technology to monitor head acceleration, head rotations and force impacts due to its positional stability.^15^ This allows more accurate data and less challenges than other potential options that have been trialled. Clothing/equipment sensors can move with respect to the skull during impact and skin-mounted sensors are prone to errors resulting from soft tissue motion.^16^ Unlike other wearable sensors, mouthguards are non-intrusive and do not interfere with an athlete's movement.

## How instrumented mouthguards work

**Fig. 1:**
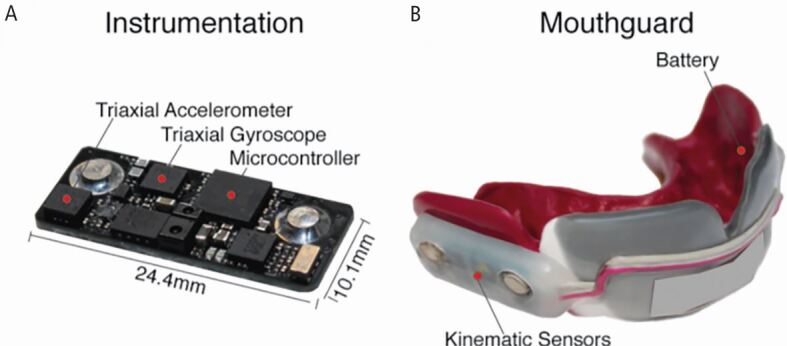
(A, B) Example of components in an iMG. Image reproduced from Kuo *et al.*, ‘Comparison of video-based and sensor-based head impact exposure,' *PLoS One*, 2018^17^

The technology involved in iMGs has three main components: the sensors, the power source and the data storage and transmission (Fig. 1).^17^

### Sensors

Mouthguards can be embedded with a variety of sensors. The integration of sensors in mouthguards allows them to capture real-time data. The most common used sensors include accelerometers, gyroscopes, and pressure sensors, which are integrated into the material of the mouthguard. They can measure linear and angular accelerations, as well as the force of impact, providing valuable information about the athlete during training or competition.^18^ Each sensor type provides specific information (Table 1).

**Table 1:** Types of sensors used

**Sensor type**	**Output**
Accelerometers	Measure the rate of change in velocity. They are critical for assessing the force and direction of impacts.
Gyroscopes	Measure rotational motion, which is important for detecting concussive impacts.
Pressure sensors	Provide data on the force applied to specific areas of the mouthguard, helping to gauge impact severity.

### Power source

A rechargeable battery is used as a power source for the technology in the iMG. Modern technology allows for the battery to be charged wirelessly. Charging is done on a regular basis. A mouthguard case can be designed for the iMG to allow ease of wireless charging. The positional placement of the battery in the mouthguard needs consideration to allow connectivity for charging.

### Data transmission and storage

The data collected by the sensors in an iMG can be downloaded after the event or transmitted wirelessly to an external device for analysis. Real time data can be transmitted via Bluetooth without the need for the iMG to be connected directly to the software after being worn. Transmitting wirelessly allows coaches and medical professionals to monitor the athletes in real-time. Some systems also include cloud-based storage, enabling longitudinal studies of an athlete's injury history and performance.

## Design of the instrumented mouthguard

The integration of the technology into the protective mouthguard provides various design challenges. Position of the technology in the mouthguard, the size of technology and the ability to charge the power supply element of the technology are important considerations when designing the iMG.

### Position of the technology

**Fig. 2:**
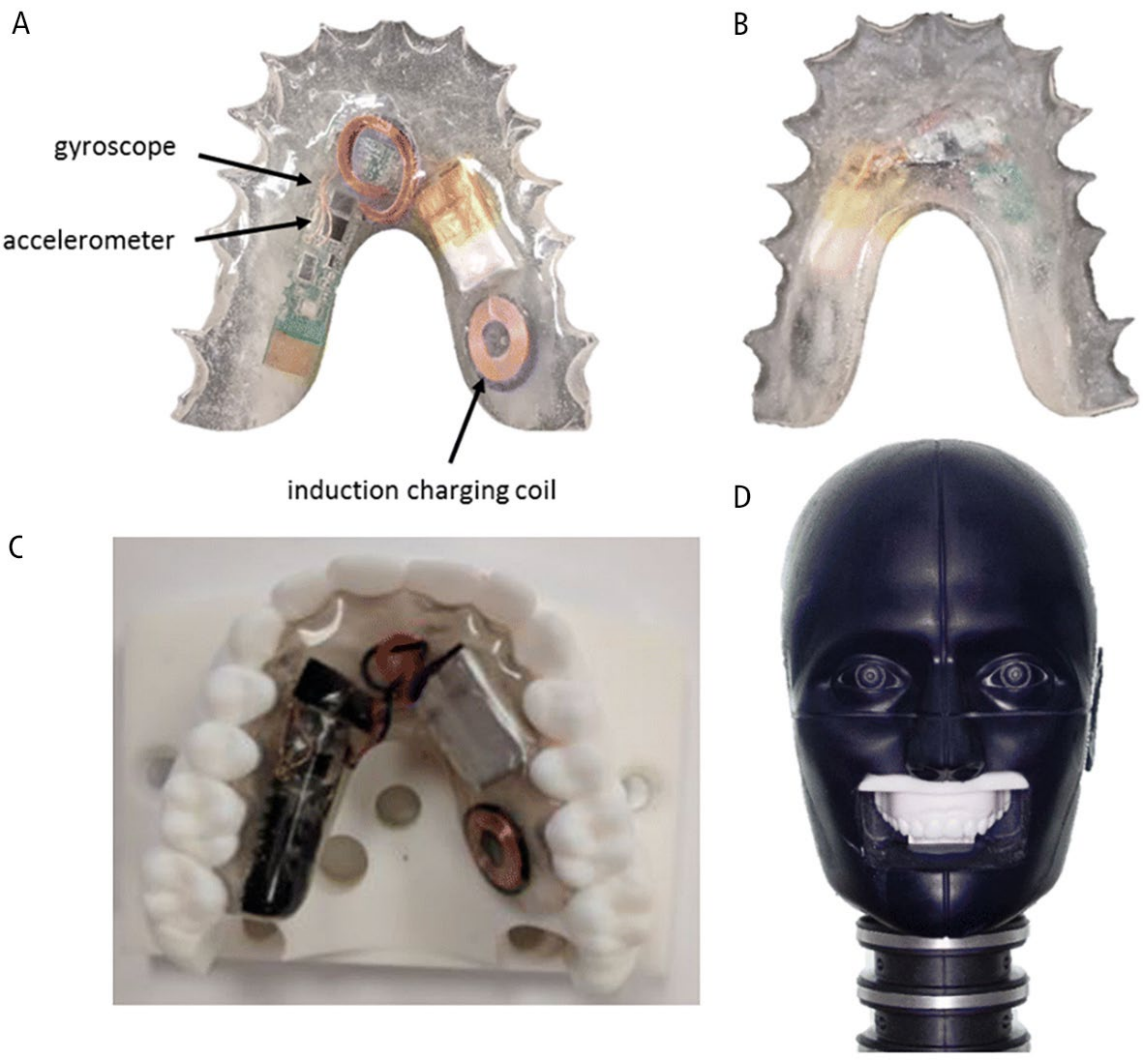
(A, B, C, D) Technology placed on palatal surface of mouthguard. Reproduced with permission from Rich *et al.*, ‘Development, Validation and Pilot Field Deployment of a Custom Mouthpiece for Head Impact Measurement', *Annals of Biomedical Engineering*, 2019, Springer Nature^21^

**Fig. 3:**
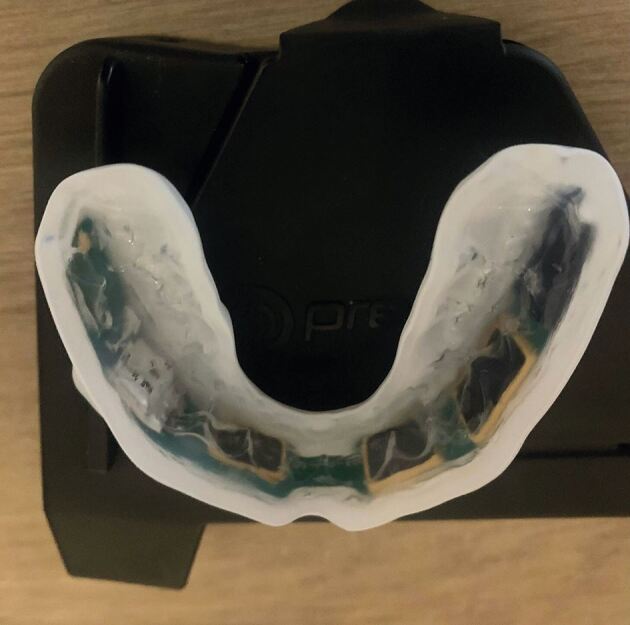
Technology placed on buccal surface of the mouthguard

Wearer comfort is important for compliance with iMG use.^19^ Some early iMG designs had positioned the technology on the palatal surface of the mouthguard (Fig. 2).^20^ This had a negative impact on wearer comfort. It is now established that the technology should be placed on the buccal surface of the mouthguard as this is more forgiving in terms of wearer comfort (Fig. 3).

Positioning the technology buccally brings in considerations regarding the protective properties of the mouthguard. The primary role of the upper sports mouthguard is to protect from orofacial trauma. A multi-layer laminated pressure thermoformed EVA mouthguard with a minimum of 3 mm in thickness in the buccal and incisal area is recommended for protection from orofacial trauma in sports.^21^ Placing technology in between the EVA layers changes the dynamics of the mouthguard. It is important to ensure adequate protection is still functioning in iMGs. There has been some anecdotal evidence suggesting iMGs without adequate EVA thickness between the teeth and the technology can increase the risk of dental trauma from a sporting impact. There also needs to be adequate EVA thickness around the technology to provide protection of electrical components from impact.^22^

### Size of technology

**Fig. 4:**
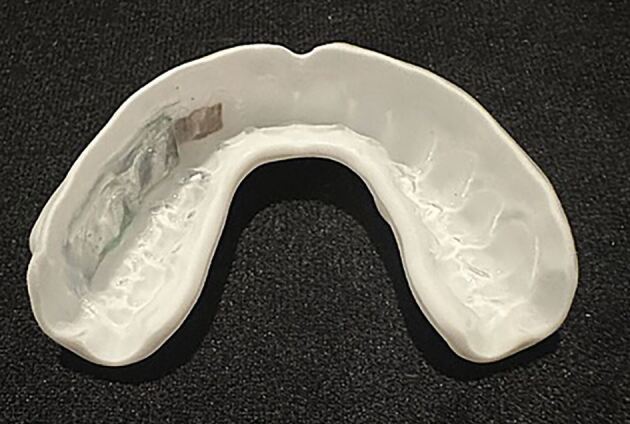
Current size of technology

Recent developments in the technology used for iMGs has seen an impressive reduction in size of the technology with the technology positioned on the buccal surface of the upper left posterior teeth (Fig. 4). This recent development has greatly improved compliance due to improved wearer comfort. ^23^

### Charging power source and data collection

Charging cases have been developed to allow wireless recharging of the battery in the iMG. This is commonly done after each session the iMG is used. The positioning of the battery in the mouthguard needs to ensure compatibility with the wireless charging case. The positioning of the sensors is important for consistent reliable data. The charging case can also allow downloading of the data collected from the iMG.

## Benefits of instrumented mouthguards in sport

Concussive impacts often involve high levels of linear and rotational acceleration, which IMGs are capable of measuring accurately.^24^^,^^25^ One of the key benefits is real-time data collection. This provides immediate feedback on an athlete's condition and performance during training or competition. iMGs can enhance concussion protocols as they assist in the identification of an HAE and allow for an accurate assessment of the forces involved. Threshold levels can be set, allowing the iMG software system to alert medical professionals, prompting them to remove the athlete from training or competition to assess for concussion symptoms.

The continuous data collected by instrumented mouthguards allow for data-driven decision-making. By analysing the data trends, injury prevention strategies can be created that are tailored to the individual needs of the athlete. These strategies may include modifications to training strategies, informing exposure windows to contact and collision in training, changes in playing style and tackle technique, or early interventions to address minor issues before they develop into more severe injuries.

## Applications of instrumented mouthguards in sport

iMGs are being used in sport to help with SRC management, performance optimisation and injury prevention. It is the use of iMGs for assessing HAEs to aid with management of SRC that has caused the biggest growth in iMG use.

### Sport-related concussion management

iMGs provide a highly accurate measurement device that could be used to complement video verification in the recognition of on-field HAEs.^26^ iMGs can provide real-time data, which allows for immediate assessment of the severity of an impact. If an athlete experiences an impact to the head, the data can indicate whether the force was high enough to warrant immediate evaluation for concussion. These devices help provide objective data to support sports medical teams with symptom assessment.

### Performance enhancement

Assessing the impact load of an athlete during training and competition can support adjustments to the training preparation to promote optimum performance during competition. Reviewing previous competition performances, a maximum limit of HAEs for the athlete is set for training to promote optimum performance during competition.

iMGs could have benefits in monitoring athlete performance. By tracking an athlete's movements and the forces exerted during play, coaches can gain insights into an athlete's technique, strength and endurance. More research is needed to quantify and understand the forces registered during hard accelerations, decelerations and cutting manoeuvres to separate this from collision and impact forces.

### Injury prevention and risk assessment

Assessing the forces exerted on an athlete's head during play could help the identification of athletes at a greater risk of injury. By tracking their performance over time, iMGs may help to better understand an individual's vulnerability to injuries. Injury prevention measures can be implemented from the feedback of the iMG data. If an athlete consistently experiences high-impact collisions, they may be advised to modify their technique or be placed under additional medical observation. Long-term use of iMGs could provide valuable data for understanding an athlete's injury history. Over time, this data could help identify patterns and correlations between the frequency and severity of impacts and the occurrence of injuries. This could lead to the development of better strategies for injury prevention and recovery.

iMGs can provide feedback on an athlete's movement and technique. Coaches can determine whether certain movements or positions correlate with a higher risk of injury and suggest corrective measures. Coaches can work with players to adjust their body positioning or improve their tackling techniques to reduce the likelihood of severe collisions.

An athlete on a return-to-play programme from injury or a SRC can benefit from analysis of the iMG data. The iMG will be able to objectively feedback if the athlete is operating at their performance level before the injury or SRC. This can help provide the athlete with confidence to return to play. iMG data may also perform a key role in preparing the brain and associated connective tissue in returning the athlete from long-term injury with a progressive overload strategy. This would allow the injured athlete to safely and incrementally return to their baseline threshold level and avoid spikes in athlete load, similar to how global positioning system software is used in return-to-running protocols for injured athletes.

iMGs are being used to assess HAEs in sports to get a better understanding of the risks associated with that sport.^27^ Military activities are being assessed with iMGs to profile a risk profile of the activity.^28^

## World Rugby mandates instrumented mouthguard use

Rugby union is a physically demanding, high-impact sport, which places significant emphasis on player safety and injury prevention.^29^^,^^30^ Recognising iMGs' potential for enhancing player safety, World Rugby mandated iMG use at elite-level rugby worldwide from 2024.^31^ This is the most significant recent application of iMGs. World Rugby is the first major sporting governing body to introduce iMG use at a significant level. Their guidelines around iMG use may become a template for other sporting governing bodies.

World Rugby require elite players to use their iMG in all training and play where a HAE might occur. Access to a head injury assessment (HIA) during a match is restricted to players who are compliant with iMG use.^31^ The first competition that World Rugby's iMG mandatory rule came into effect was the 2024 Men's Six Nations Competition with Scottish player George Turner becoming the first player to be removed from the field of play for a HIA due to feedback from an iMG.^32^

World Rugby has developed minimum specifications for iMGs using best practice validity assessments methods, and expert consultation with engineers and biomechanists experienced in head sensors (Table 2).^33^ In all uses in rugby there are minimum specifications for impact energy absorption and laboratory and on-field testing, with additional specifications for real-time performance for competitions where the HIA process is used. Due to the relatively new and evolving nature of iMG technology, World Rugby has committed to reviewing their specifications every six months to keep up to date with iMG technology's evolution and limitations.

**Table 2:** World Rugby instrumented mouthguard specifications

**Performance specification**	**Required performance specification**	**Recommended performance specification**	**Required for**
Impact energy absorption	CE certification from a notified body		All levels and age grades of the game
Laboratory testing	When testing peak linear acceleration, peak rotational acceleration, and peak rotational velocity at least 80% of measurements must have less than 20% error, must have a proportional bias of 10% or less (slope between 0.90 and 1.10), and a concordance correlation coefficient value of 0.90 or higher	When testing peak linear acceleration, peak rotational acceleration, and peak rotational velocity at least 86% of measurements have less than 20% error, must have proportional bias of 3% or less (slope between 0.97 and 1.03), and a concordance correlation coefficient value of 0.96 or higher	All levels and age grades of the game
On-field testing	When tested in the field the: Positive predictive value must be ≥90 Sensitivity (to detect HAEs) must be ≥80%		All levels and age grades of the game
Real-time performance	Provide data on the number and magnitude of HAEs from the first-half or second half within five minutes of the respective half. Data must be within 10% of downloaded postprocessed data, after any inbuilt processing has been applied. Inbuilt processing may take place on-device or in the cloud. In this instance, ‘real-time' refers to half-time and end-of-match access to the data on the pitchside device portal.	Data on the number and magnitude of HAEs can be accessed within 30 seconds of the event. After any inbuilt processing has been applied on-device, the data access on the pitchside portal must be within 10% of downloaded postprocessed data. In this instance, ‘real-time' refers to instantaneous (within 30 seconds) access to the data on the pitchside device portal.	Competitions where the HIA process is used
HAEs, head acceleration events; HIA, head injury assessment			

In rugby competitions where an HIA process is used, a player will be removed from the field of play for an HIA if the iMG signals the player has experienced HAEs that have been over a set threshold level. The threshold level can be meet by a single HAE or accumulation of HAEs. Recent research suggests that this will happen once every 60–100 min in men and 200–300 min in women.^34^

World Rugby iMG guidelines state that manufacturers have a responsibility to ensure that any product being sold or supplied for use in rugby has been previously tested by a recognised World Rugby Accredited Test Institute to ensure it meets World Rugby specification requirements.^33^ This is due to the varying levels of performance from commercially available iMG products.^35^ Table 3 shows the current (April 2025) approved iMGs by World Rugby.

**Table 3:** Approved instrumented mouthguards for use in rugby union

**Manufacturer**	**Model**
Prevent Biometrics	Prevent Version 1.4 Custom iMG
Prevent Biometrics	Prevent Version 2.0 Custom iMG
HITIQ Ltd	NEX-MG-1-0004
HITIQ Ltd	NEX-MG-1-0003
**Approved for use in premium-level-HIA matches**	
**Manufacturer**	**Model**
Prevent Biometrics	Prevent Version 1. 4 Custom iMG
Prevent Biometrics	Prevent Version 2. 0 Custom iMG
iMG, instrumented mouthguards; HIA, head injury assessment	

## Challenges and limitations of instrumented mouthguards

Despite their numerous benefits, iMGs also face several challenges and limitations. iMGs with the accompanying software and analysis tools are more expensive than traditional mouthguards, which will make them inaccessible to some athletes or sports programmes, especially at the youth or amateur levels. Therefore, player welfare equity will be an issue.

Another challenge is ensuring the accuracy and reliability of the data collected. Despite the advancements in sensor technology, the accuracy of the data collected by iMGs can be compromised by factors such as the placement of sensors, the athlete's mouthguard fit, mechanical forces (biting, clenching or impact forces) and environmental conditions (temperature, moisture contamination and electromagnetic interference). Ensuring the reliability of the data under different circumstances remains a challenge for developers.^36^ The sensors embedded in the mouthguards must be calibrated correctly to ensure that the measurements reflect the actual forces experienced by the athlete.^37^ Any errors in data transmission or sensor malfunction could compromise the integrity of the results.^38^ Sensors in isolation to confirm head impacts have been shown to have high occurrences of false positives resulting in an overestimation of head impact exposure due to the sensitivity and inaccuracies of the sensor used.^39^ Data should be interpreted in context and supported using video analysis if available.

Given the high-impact nature of many sports, ensuring the durability of the sensors and the mouthguard material is essential. Regular maintenance and checking of the equipment may also be necessary to maintain consistent performance.

iMGs need to be comfortable to wear for ensuring athlete compliance.^19^ The design of iMGs needs to consider this important aspect. Developments in design and the size of the technology have helped improve iMG comfort. Some athletes find it difficult to wear a protective mouthguard with comfort and a perceived impact on breathing performance being the issues reported, making it challenging for iMG implementation and use.^19^

The collection of information on athlete performance and health has ethical concerns absent from traditional mouthguards. The sensitive nature of concussion and injury data means that measures must be in place to protect athletes' personal information. There could be challenges at youth and amateur levels. Ensuring that data are not misused or improperly accessed is essential and measures put in place to provide data privacy and security.^40^

Implementation of iMGs to a team requires dedicated knowledgeable support staff. Time constraints of staff and lack of understanding of data are barriers to iMG use. Continued education regarding the use and application of iMG data are required.^19^

The management and analysis of iMG data are complex and resource-intensive which demands significant time and technical expertise. Analysing data from iMGs involves data cleaning, synchronisation with video footage, artefact detection, and event classification.^41^ This imposes financial and time constraints. The development of streamlined and standardised analysis protocols along with the possibility of machine learning will be essential to manage the growing volume of data effectively.^42^

## The future of instrumented mouthguards

The future of instrumented mouthguards looks promising, with advances in sensor technology and data analytics likely to drive further innovation in this field.

### Smaller, more advanced sensors

Future mouthguards may feature smaller, more advanced sensors that offer even greater precision and accuracy in measuring impacts. As sensor technology advances, mouthguards may become even lighter, thinner, and more comfortable without sacrificing performance or durability.

### Enhanced sensor capabilities

As sensor technology continues to evolve, future iGMs may include multiple features, such as coupling HAE sensors with sensors for monitoring bioelectrical signals like heart rate and muscle activation, further enhancing the ability to assess an athlete's physical condition.

### Integration with other wearables technologies

iGMs could be integrated with other wearable technologies, such as smart clothing or wristbands, to provide a more comprehensive picture of an athlete's health and performance.

### Reduction in healthcare costs

While the initial cost of an iGM may be higher than that of a traditional mouthguard, the potential to reduce healthcare costs by preventing injuries and monitoring recovery could result in long-term savings.

### Widespread adoption in youth sports

The benefits of iMGs are particularly evident in youth sports, where the risk of head injuries is high. As technology becomes more affordable and accessible, iGMs could become a standard piece of equipment for young athletes, helping to prevent injuries and promote safer play.

### Graduated return-to-play protocols

As more is understood around the magnitude of specific impacts at differing speeds, the iMG may form part of a data-informed graded exposure in return-to-play protocols.

## The sports dentist's role in iMG use in sport

The role of the sports dentist has been undervalued in iMGs use in sport. In the early development stages of iMGs, dentist involvement was limited as researchers and companies used a boil and bite mouthguard to house the technology. The importance of the mouthguard part of the iMG was underappreciated. This mindset has changed, with the leading international iMG products involving custom-fitted laminated pressure thermoformed mouthguards. This has developed from a better understanding of how the fit and design of the iMG impacts the device safety and data accuracy.

Dentists are providing support with digital impressions of athletes to start the iMG production process. There is great benefit to have a dentist deliver the iMG to the athlete. Adjustments can be made to the iMG by the dentist to ensure the fit, comfort, and safety of the device is specific to that athlete.

The role of the sports dentist could develop to providing support with iMG use in management of SRC during training and competition, management and analysis of the data, and direct provision of iMGs to athletes. As sensors and technology develop, iMGs may have the ability to monitor risk of oral diseases/conditions such as caries, erosive tooth wear (e.g., salivary pH and buffering) and periodontal diseases (e.g., microbiome/bacterial metabolite assessment). This is important as elite and professional sport has high levels of oral diseases. Such rich data could provide novel insights into aetiology and prevention with potential for translation to other ‘high risk' population groups. It is therefore important that sports dentists stay up to date with the development of iMG use in sport and build relationships with other professionals involved in this area.

## Conclusion

iMGs represent a significant leap forward in the integration of technology and athlete safety. By combining the traditional protective functions of mouthguards with cutting-edge sensors that measure impact forces and HAEs, iMGs provide real-time, data-driven insights that are valuable for monitoring concussion risk, injury prevention and performance enhancement. The mandatory adoption of iMGs in elite rugby by World Rugby from 2024, sets a potential precedent for other sports organisations. The minimum specifications, guidelines on use and the protocols from the data analysis created by World Rugby could become the international standard for iMG use for assisting with SRC. While challenges such as cost, data reliability, and comfort remain, the ongoing advancements in sensor technology, as well as the growing understanding of their benefits, suggest an evolving future for iMGs. As the technology becomes more affordable and accessible, it could play a pivotal role in safeguarding athletes at all levels.
